# Olfactory Impairment in Parkinson’s Disease Patients with Tremor Dominant Subtype Compared to Those with Akinetic Rigid Dominant Subtype: A Pilot Study

**DOI:** 10.3390/brainsci12020196

**Published:** 2022-01-31

**Authors:** Paolo Solla, Carla Masala, Tommaso Ercoli, Gianni Orofino, Francesco Loy, Ilenia Pinna, Laura Fadda, Giovanni Defazio

**Affiliations:** 1 Department of Neurology, University of Sassari, Viale S. Pietro 10, 07100 Sassari, Italy; psolla@uniss.it; 2 Department of Biomedical Sciences, University of Cagliari, SP 8 Cittadella Universitaria, 09042 Monserrato, Italy; floy@unica.it (F.L.); ilenia.pinna.1994@gmail.com (I.P.); 3 Movement Disorders Center, Department of Neurology, University of Cagliari, SS 554 km 4.500, 09042 Cagliari, Italy; ercolitommaso@me.com (T.E.); dr.g.orofino@gmail.com (G.O.); fadda_laura@yahoo.it (L.F.); giovanni.defazio@unica.it (G.D.)

**Keywords:** neurodegenerative disorders, neurodegeneration, movement disorders, Parkinson’s disease, olfactory dysfunction

## Abstract

Background: Parkinson’s disease (PD) may present different motor subtypes depending on the predominant symptoms (tremor or rigidity/bradykinesia). Slower disease progression and cognitive decline were observed in tremor-dominant (TD) patients compared to those with the akinetic-rigid dominant (ARD) subtype. Although olfactory dysfunctions are well-known disturbances in PD patients, correlations among PD different subtypes and olfactory impairment were not clearly studied. Thus, we investigated the possible olfactory impairment in PD patients with TD and ARD subtypes as compared to healthy controls. Methods: A sample of 132 participants were enrolled: 62 PD patients divided into ARD (*n* = 42) and TD (*n* = 20) subgroups using tremor/rigidity ratio, and 70 healthy controls. Olfactory function was assessed with the Sniffin’ Sticks Test. Results: Odor threshold was significantly lower in the ARD than in the TD subtype, while odor identification, discrimination scores, and their sum (TDI score) were not significantly different. On multivariate linear regression analysis, the tremor/rigidity ratio was a significant predictor of odor threshold. Conclusions: Our pilot study showed a significant olfactory dysfunction in PD patients with the ARD subtype. This evidence confirms the biological relevance of clinical subgroups in PD patients, suggesting the existence of a different pathophysiological mechanism between the ARD and TD clinical subtypes.

## 1. Introduction

Parkinson’s disease (PD) is a chronic neurodegenerative disorder characterized by motor symptoms (MSs) and non-motor symptoms (NMSs) [1]. Bradykinesia, along with tremors, rigidity, and postural instability, are the main common MSs, while among the NMSs are cardiovascular disorders, sleep, cognitive impairment, and visual, taste, and olfactory dysfunctions [2]. The akinesia and bradykinesia may be due to decreased dopamine activity in the dorsal striatum and globus pallidus.

Based on the most prominent motor sign, patients were classified into different clinical subtypes with a diverse prognosis and course: akinetic-rigid-dominant (ARD) and tremor-dominant (TD) [3,4,5]. TD subtypes were associated with an impairment in basal ganglia and in the cerebellar–thalamic pathway [6]. The ARD subtypes were associated with a damage in the mesolimbic cortex [7,8] and may partially overlap with the Progressive supranuclear palsy-Parkinsonism predominant [9].

In addition, previous studies suggested that the ARD and TD subtypes in PD patients may have a different clinical course with slower disease progression and mild cognitive decline in TD patients compared to those with the ARD subtype [3,10,11]. In the same way, these subtypes may also induce different responses to therapy and to the possible development of the cardiovascular side effects of dopaminergic drugs among PD patients [12]. Another study evaluated the differences in the frontal tasks and behavior between the two subtypes, suggesting that patients with TD were more depressed and anxious, while those with the ARD subtypes were more prone to apathy and irritability [13].

Among NMSs in PD, olfactory dysfunction is considered the most common with a prevalence of 95% [14,15,16], but the possible correlations with different PD subtypes have not been understood. A previous study evaluated only odor identification using an UPSIT test, reporting that patients with TD had significantly better odor identification scores compared to those with ARD [4].

In this context, in order to advance the comprehension of the neural mechanism involved in the clinical manifestation of these subtypes, our aim was to investigate odor threshold, odor discrimination, and odor identification in PD patients with the TD and ARD subtypes compared to healthy controls.

## 2. Materials and Methods

In this study, 132 participants were evaluated (66 men and 66 women), including 62 PD patients (mean age ± SD; 72.39 ± 8.99) and 70 healthy controls (mean age ± SD; 70.64 ± 8.14). PD patients were consecutively recruited at the Movement Disorders Center of the University of Cagliari during regular out-patient follow-up visits. All patients had a PD diagnosis based on the Postuma criteria [17] performed by a neurologist specialized in movement disorders.

Participants with cognitive impairment or with any disorder interfering with olfactory evaluation, such as a history of head or neck trauma, stroke, atypical parkinsonism, psychiatric conditions, or chronic/acute rhinosinusitis of the disease, were excluded. All these exclusion criteria were verified by an expert neuropsychologist. The demographic and clinical information of all participants, including age, sex the age at PD onset, and current medications, was collected. The age at onset was determined as the age at which the patient had first observed initial motor symptoms of parkinsonism. The assessments were carried out in all recruited patients after receiving their usual medication. The levodopa equivalent daily dose (LEDD) was computed as previously reported [18]. Motor impairment was assessed by the mean of the Modified Hoehn and Yahr (HY) Scale [19] and the Unified PD Rating Scale (UPDRS) part III [20]. Patients were classified based on established methods into the TD or ARD subtypes using items from the UPDRS part III [20]. We defined the clinical subgroups TD and ARD for each patient in a manner similar to Lewis and Colleagues [21]. The tremor score was calculated from the sum of UPDRS items 20 and 21 divided by 7. The non- tremor score was calculated from the sum of UPDRS items 18, 19, 22, 27, 28, 29, 30, and 31, divided by 12. PD patients were grouped in the TD subtype if the tremor/non tremor score ratio was equal to or greater than 1, and in the ARD subtype if the tremor/non tremor score ratio was less than 1. Cognitive abilities were evaluated using the Montreal Cognitive Assessment (MoCA), which consists of different domains: visual-constructional skills, executive functions, attention and concentration, memory, language, conceptual thinking, calculations, and spatial orientation [22,23,24]. The MoCA total score was 30, and any score ≥ 26 was considered normal.

Fatigue was evaluated by the Parkinson’s Disease Fatigue Scale (PFS) [25] and apathy was assessed by the Starkstein Apathy Scale (SAS) [26].

Olfactory function was assessed by the Sniffin’ Sticks Extended Test (Burghart Messtechnik, Wedel, Germany), which consists of three different parameters: odor threshold, odor discrimination, and odor identification [27,28,29,30,31,32]. All participants were instructed to drink only water 1 h before the test, and to avoid any smoking and scented products on the testing day.

The Sniffin’ Sticks are pen-like odor-dispensing devices, and the complete procedure lasted 30–40 min [33]. Patients were blindfolded during the odor threshold and odor discrimination tasks. First, odor threshold was determined with 16 stepwise dilutions of n-butanol [34]. A three-alternative forced-choice task (3AFC) and the single-staircase technique were used [29,30,31,32]. Scores of odor threshold ranged from 1 to 16. Secondly, odor discrimination was assessed using 16 trials. Three different pens were presented using the 3AFC task; two contained the same odor (non-target odors) and the third presented a different odor (target odor). The odor discrimination score was calculated as the sum of the correct responses and ranged from 0 to 16 points [31,32]. Finally, odor identification was evaluated using 16 common odors presented with four verbal descriptors in a multiple forced-choice format (one target and three distractors) and score was calculated as the sum of the correct responses and ranged from 0 to 16 points. The TDI (Threshold-Discrimination-Identification) score represents the sum of the odor detection threshold, discrimination, and identification scores. Values of TDI > 30.5, ≤30.5, and ≤16.5 were considered normosmia, hyposmia, and functional anosmia, respectively [28,35].

At first, a sample size calculation was performed in order to assess the required minimum number of subjects to be enrolled in the study. Based on previous studies using similar protocols [29,31], it is expected that a total of 120 subjects was considered adequate in order to detect the investigated differences. A power calculation based on comparable studies and considering a critical effect size f = 0.3 (a medium effect), with 85% power and a 5% significance level in a standard one-way ANOVA, suggested a required minimum number of 120 total subjects, and a power calculation considering a critical effect size f2 = 0.15 (a medium effect), with 85% power and a 5% significance level for each investigated factor in a multiple linear regression model, suggested a required minimum number of 120 total subjects (G-Power 3.1).

Statistical analysis was performed by the SPSS software version 24 for Windows (IBM, Armonk, NY, USA). Normal distribution of the data was assessed using the Shapiro-Wilk test. Statistical differences between the healthy controls and the patients divided between TD and ARD were performed using one-way analyses of variance (ANOVA) with the Bonferroni correction for multiple comparisons or the chi-square (χ^2^) statistic with the Yates correction, as appropriate. After the ANOVA test, pairwise *t*-tests were conducted in order to calculate differences between each group.

In order to identify the more promising factors for the multivariate linear regression analysis, Pearson’s rank correlation coefficients (r) were computed, evaluating PD patients’ relations between the tremor/rigidity ratio versus sex, age, HY, UPDRS, LEDD, cognitive abilities (MoCA), and olfactory function.

Moreover, a multivariate linear regression analysis was performed to assess the contribution of the odor threshold, sex, and age on the tremor/rigidity ratio. In the multivariate linear regression analysis, the tremor/rigidity ratio was a dependent variable, while age, sex, and odor threshold were independent variables. Data were presented as mean values ± standard deviation. Significance was set at the 0.01 level after the Bonferroni correction. This study was approved by the local Ethics Committee (Prot. PG/2018/10157) and performed according to the Declaration of Helsinki. Participants received an explanatory statement and gave their written informed consent to participate to the study.

## 3. Results

One hundred and thirty-two participants were enrolled: 20 PD patients with the TD subtype, 42 with the ARD subtype, and 70 age-matched healthy controls. The global demographic and clinical characteristics of patients with the TD and ARD subtypes and healthy controls are summarized in Table 1.

The mean age was 73.4 ± 8.9 in PD patients with the ARD subtype, 70.4 ± 9 in those with the TD subtype, and 70.6 ± 8.1 in the healthy controls. No significant differences were found for age, weight, and height between all groups (*p* = 0.240, *p* = 0.628, and *p* = 0.224, respectively).

Patients with TD subtypes were not statistically different for the age at PD onset and for disease duration compared to those with ARD subtypes (*p* = 0.439 and *p* = 0.436, respectively). Mean values ± standard deviation of the age at PD onset were 64.4 ± 8.4 and 67.5 ± 10.5 in the TD and ARD subtypes, respectively. Similarly, no significant differences were observed between the TD and ARD subtypes for HY, UPDRS, and LEDD (*p* = 0.401, *p* = 0.261, and *p* = 0.521, respectively). In patients with the TD subtype, the mean values ± standard deviation were 2.2 ± 0.8 for HY, 20.3 ± 13.2 for the UPDRS score and 257.9 ± 222.3 for LEDD. In patients with ARD subtypes, the mean values were 2.3 ± 0.7, 24.6 ± 14.1, and 301.42 ± 252.58 for HY, UPDRS, and LEDD, respectively (Table 1).

In regards to cognitive abilities (MoCA), the mean scores ± standard deviation were 23.2 ± 3.5, 21.6 ± 5.5, and 24.6 ± 3.3 in the TD and ARD subtypes and the healthy controls, respectively. Significant differences were found between these three groups (F_(2, 129)_ = 6.905, *p* = 0.003, partial η^2^ = 0.097). In pairwise *t*-tests, patients with ARD showed a significant decrease in MoCA score compared to the healthy controls (*p* = 0.001). For apathy, the mean scores ± standard deviation were 9.9 ± 6.7, 12.87 ± 6.4, and 9.6 ± 5.4 for the TD and ARD subtypes compared to the healthy controls. Although, the fatigue mean scores ± standard deviation were 2.8 ± 0.7, 3 ± 0.9, and 2.1 ± 0.8 in patients with the TD and ARD subtypes compared to the healthy controls. Patients with the ARD subtype showed a significant increase in apathy and fatigue compared to those with TD and to the healthy controls (F_(2, 77)_ = 24.25, *p* = 0.001 and F_(2, 77)_ = 49.97, *p* = 0.001) (Table 1).

Moreover, for the odor threshold, the mean values ± standard deviation were 3.4 ± 2.7, 1.9 ± 1.4, and 6.3 ± 4.1 in patients with the TD and ARD subtypes compared to the healthy controls (Table 2). A significant difference was observed for odor threshold between the three groups (F_(2, 129)_ = 26.61, *p* < 0.001, partial η^2^ = 0.289). In particular, in pairwise analyses, patients with the TD and ARD subtypes showed a significant impairment in odor threshold compared to the healthy controls (*p* < 0.001). In addition, patients with the ARD subtypes exhibited a significant impairment in odor threshold compared to those with TD (*p* = 0.0084). Similarly, for odor discrimination, the mean values ± standard deviation were 8.6 ± 2.2, 7 ± 3.1, and 11.2 ± 2.4 in the TD and ARD subtype patients compared to the healthy controls (Table 2). A one-way, between subjects ANOVA showed significant differences for odor discrimination between all three groups (F_(2, 129)_ = 35.477, *p* < 0.001, partial η^2^ = 0.355). In pairwise analyses, patients with the TD and ARD subtypes showed a significant decrease in odor discrimination score compared to the healthy controls (*p* < 0.001). No significant differences were observed in odor discrimination between PD patients with the ARD subtype compared to those with TD.

In odor identification, the mean values ± standard deviation were 8.3 ± 3.1, 7.1 ± 3.4, and 11.9 ± 2.1 in the TD and ARD subtype patients and in the healthy controls, respectively (Table 2). In the one-way, between subjects ANOVA significant differences were observed between all three groups (F_(2, 129)_ = 44.864, *p* < 0.001, partial η^2^ = 0.410). Significant differences in odor identification score were observed only when patients with the TD and ARD subtypes were compared to the healthy controls, showing a significant decrease (*p* < 0.001), while no significant differences (*p* = 0.186) were found between the TD and ARD subtype patients.

The mean values ± standard deviation for TDI score, which represents the global olfactory function, were 20.3 ± 6.1, 16 ± 6.5, and 29.4 ± 5.7. In the one-way, between subjects ANOVA significant differences were observed between all three groups (F_(2, 129)_ = 68.735, *p* < 0.001, partial η^2^ = 0.516). In pairwise analyses, PD patients with the TD and ARD subtypes showed a significant decrease in TDI score compared to the controls (*p* < 0.001). No significant differences were found between patients with the ARD subtype compared to those with TD in global olfactory score (TDI) (*p* = 0.017). Among PD patients with the TD subtype, 75% (*n* = 15) of subjects were observed with hyposmia and 25% (*n* = 5) with anosmia, while among those with ARD, 50% (*n* = 21) of patients showed hyposmia and 50% (*n* = 21) showed anosmia. Among PD patients, no significant differences were observed in the frequency of hyposmia and anosmia between the TD and ARD subtypes (Yates χ^2^ = 0.878, *p* > 0.05 and χ^2^ = 1.528, *p* > 0.05, respectively).

**Figure 1:**
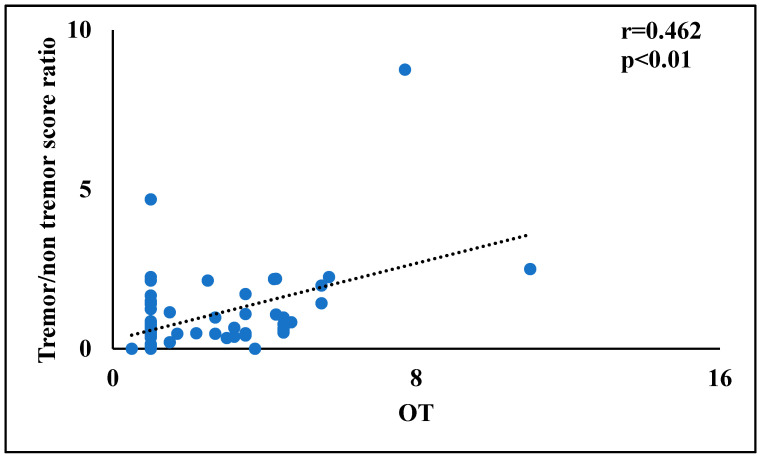
A scatterplot of the relationship between the tremor/rigidity ratio versus the odor threshold in patients with Parkinson’s disease.

**Figure 2:**
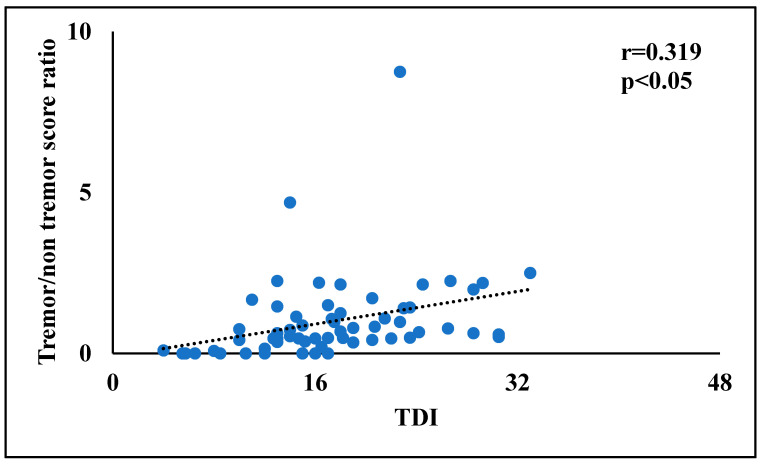
A scatterplot of the relationship between the tremor/rigidity ratio versus the odor threshold, discrimination, and identification (TDI) score in patients with Parkinson’s disease.

Finally, in order to identify the more promising factors for the multivariate linear regression analyses, bivariate correlations were performed between tremor/rigidity ratio versus motor and non-motor symptoms. In PD patients, the tremor/rigidity ratio was significantly correlated to odor threshold (r = 0.464, *p* < 0.01) (Figure 1) and TDI score (r = 0.319, *p* < 0.05) (Figure 2). No significant correlations were observed between the tremor/rigidity ratio versus sex (r = −0.106, *p* > 0.05), age (r = −0.024, *p* > 0.05), HY (r = −0.214, *p* > 0.05), UPDRS (r = 0.115, *p* > 0.05), LEDD (r = −0.113, *p* > 0.05), odor discrimination (r = 0.277, *p* > 0.05), odor identification (r = 0.151, *p* > 0.05), or cognitive abilities (MoCA) (r = 0.087, *p* > 0.05).

In addition, a multivariate linear regression analysis was performed using the tremor/rigidity ratio as a dependent variable, while the independent variables were odor threshold, age, and sex. A significant contribution emerged for odor threshold (F_(5, 58)_ = 5.944, *p* = 0.001), while no significant contributions were found for age or sex. This model explains the 20% of variance (Adjusted R^2^ = 0.196) (Table 3).

## 4. Discussion

Previous studies suggested the existence of motor subtypes among PD patients with different clinical patterns and possible distinct pathogenic pathways [3,5,36]. PD motor subtypes were predominantly divided into the TD and ARD subtypes [3], with pathological differences between these motor subtypes [37]. Indeed, patients with the TD subtype are characterized by slower disease progression, a mild level of cognitive impairment, and a minor frequency of neuropsychiatric disorders compared to those with the ARD subtype [21,38]. In particular, patients with the ARD subtype exhibited an impairment in the neural pathway of the mesolimbic cortex, while the TD subtype showed severe impairment in cerebellar thalamic projections [8]. Moreover, PD patients with the TD subtype showed a slower impairment in cardiovascular autonomic modulation in comparison to those with the ARD subtype [12], suggesting a possible different response to the development of the cardiovascular side effects of dopaminergic drugs.

Few specific studies evaluated the differences in olfactory function between the TD and ARD subtypes [4,39,40,41], although the clear difference between the ARD and TD subtypes in olfactory dysfunction is still not clearly known.

Moreover, olfactory dysfunction is considered one of the most common NMSs in PD and usually precedes the presence of clinical motor symptoms [20,42,43]. The potential causes of olfactory impairment are still unknown, but many hypotheses have been proposed. Previous studies indicated that olfactory dysfunction may be related to an impairment in olfactory bulb volume, in the pyriform cortex, and in the orbitofrontal cortex [44]. Other previous studies indicated no significant differences in olfactory bulb volume between PD patients compared to healthy controls [45,46,47].

In this context, our findings suggested a significant difference in olfactory impairment between PD patients with the TD subtype in comparison to those with the ARD subtype. Patients with TD subtypes showed a better olfactory function (odor threshold) compared to those with ARD subtypes, although no clear differences in odor identification, odor discrimination, and TDI score were identified. In fact, previous studies reported the highest UPSIT scores in TD patients compared to those with the ARD subtype [4,39], but differently from these studies, which used the only UPSIT to assess olfactory impairment, we preferred the Sniffin’ Sticks Extended Test, which permits not only the simple evaluation of odor threshold, but also the assessment of odor discrimination and identification. Indeed, the odor threshold is usually considered associated to the nasal epithelium and individual differences of the nasal cavity, while the odor discrimination and the odor identification are usually associated to central pathways such as the orbitofrontal cortex, the piriform cortex, and the amygdala [34].

Thus, we observed that the tremor/rigidity ratio was significantly correlated only with the odor threshold, while no significant correlations were observed versus odor discrimination and odor identification.

In this scenario, we hypothesized that differences in olfactory disfunctions among the TD and ARD subtypes are more likely related to peripheral damage than to a different impairment of the central pathways. However, the central pathways are not unaffected in PD patients with the ARD and TD subtypes, as evidenced by the strong impairment in odor discrimination and identification in comparisons to control subjects.

These results suggested that the pathological neurodegeneration in patients with the ARD subtype may involve two different pathways, as postulated in previous studies [40,41]. In particular, the TD subtype may involve damage in the basal ganglia and in the cerebello-thalamo-cortical circuit [40], while the ARD subtype showed severe impairment in striatal-thalamo-cortical and in cerebello-thalamo-cortical circuits [41].

Regarding the differences in dopamine levels between the two subtypes, patients with the ARD subtype had lower level of dopamine in the globus pallidus compared to those with TD [48]. In addition, our data showed that patients with ARD subtypes showed higher scores in apathy compared to those with TD subtypes.

Our results, in line with a previous study [5], suggested that patients with ARD were more prone to apathy and depression. In our data, no significant differences were observed between the TD and ARD subtypes for age, sex, disease duration, UPDRS, HY, LEDD, and the age of onset.

This study has some limitations. The small sample size requires further studies to confirm our findings. A bias in case selection was unlikely because the demographic and clinical features of our patients were consistent with those typically observed in PD.

## 5. Conclusions

Despite the foregoing limitations, our pilot study showed a more evident impairment of odor threshold in PD patients with the ARD subtype. Such evidence supports the biological relevance of clinical subtypes in PD patients, suggesting the concept of a different pathophysiological process between these different clinical forms.

## Figures and Tables

**Table 1:** The demographic and clinical characteristics of Controls and PD subjects with the tremor-dominant (TD) and akinetic-rigid dominant (ARD) subtypes. Data are expressed as N (%), mean ± SD.

	TD Subtype	ARD Subtype	Controls	*p*-Value		
				C/TD	C/ARD	TD/ARD
Demographics	N = 20	N = 42	N = 70			
Sex N (% female)	9 (45.0%)	19 (45.2%)	38 (54.3%)	NS	NS	NS
Age	70.4 ± 9.0	73.4 ± 8.9	70.64 ± 8.1	NS	NS	NS
Height (cm)	167 ± 9.8	162 ± 6.9	163 ± 10.3	NS	NS	NS
Weight (kg)	71.4 ± 12.6	72.2 ± 15	69.3 ± 17.3	NS	NS	NS
Age at PD onset (years)	64.4 ± 8.4	67.5 ± 10.5	NA	NA	NA	NS
PD duration (years)	9.2 ± 19.1	5.8 ± 4.5	NA	NA	NA	NS
Hoehn and Yahr	2.2 ± 0.8	2.3 ± 0.7	NA	NA	NA	NS
UPDRS	20.3 ± 13.2	24.6 ± 14.1	NA	NA	NA	NS
LEDD	257.9 ± 222.3	301.4 ± 252.5	NA	NA	NA	NS
MoCA	23.2 ± 3.5	21.6 ± 5.5	24.6 ± 3.3	NS	**	NS
Apathy	9.9 ± 6.7	12.9 ± 6.4	9.6 ± 5.4	NS	***	***
Fatigue	2.8 ± 0.7	3 ± 0.9	2.1 ± 0.8	NS	***	***

Legend: LEDD = levodopa equivalent daily dose; MoCA = Montreal Cognitive Assessment; N = number of participants; PD = Parkinson’s disease; SD = standard deviation; NA = not available; C = controls; ARD = akinetic rigid dominant subtype; TD = tremor dominant subtype; UPDRS = Unified Parkinson’s Disease Rating Scale. NS = *p* > 0.05; ** = *p* ≤ 0.01; *** = *p* ≤ 0.001. Statistical differences were performed using pairwise *t*-tests and the Bonferroni correction.

**Table 2:** The olfactory function in PD patients with the tremor-dominant (TD) (*n* = 20) and in those with the akinetic-rigid dominant (ARD) (*n* = 42) subtypes compared to the controls (*n* = 70). Data are expressed as mean ± SD.

Sniffin’ Stick Test	TD Subtype (*n* = 20)	ARD Subtype (*n* = 42)	Controls (*n* = 70)	*p*-Value		
				C/TD	C/ARD	TD/ARD
Odor threshold	3.4 ± 2.7	1.9 ± 1.4	6.3 ± 4.1	**	****	**
Odor discrimination	8.6 ± 2.2	7 ± 3.1	11.2 ± 2.4	****	****	NS
Odor identification	8.3 ± 3.1	7.1 ± 3.4	11.9 ± 2.1	****	****	NS
TDI score	20.3 ± 6.1	16 ± 6.5	29.4 ± 5.7	****	****	NS

Legend: TDI = threshold–discrimination–identification scores; SD = standard deviation; C = controls; ARD = akinetic rigid dominant subtype; TD = tremor dominant subtype. NS = *p* > 0.05; ** = *p* ≤ 0.01; **** = *p* ≤ 0.0001. Statistical differences were performed using pairwise *t*-tests and the Bonferroni correction.

**Table 3:** Multivariate linear regression analyses in patients with Parkinson’s disease using the tremor/rigidity ratio as a dependent variable.

	Unstandardized Coefficients		Standard Coefficients		
	B	Std Error	β	*t*	Significance
Odor threshold	0.308	0.075	0.475	4.121	***
Sex	−0.375	0.306	−0.143	−1.224	NS
Age	0.002	0.017	0.016	0.138	NS

Legend: NS = *p* > 0.05; *** = *p* ≤ 0.001.

## Data Availability

Not applicable.
